# Neuroradiologic Evaluation of MRI in High-Contact Sports

**DOI:** 10.3389/fneur.2021.701948

**Published:** 2021-08-11

**Authors:** Derek McAllister, Carolyn Akers, Brian Boldt, Lex A. Mitchell, Eric Tranvinh, David Douglas, Maged Goubran, Jarrett Rosenberg, Marios Georgiadis, Mahta Karimpoor, Phillip DiGiacomo, Nicole Mouchawar, Gerald Grant, David Camarillo, Max Wintermark, Michael M. Zeineh

**Affiliations:** ^1^Department of Radiology, Stanford School of Medicine, Stanford, CA, United States; ^2^Department of Radiology, Uniformed Services University of the Health Sciences, Bethesda, MD, United States; ^3^Department of Radiology, Madigan Army Medical Center, Tacoma, WA, United States; ^4^Hawaii Permanente Medical Group, Honolulu, HI, United States; ^5^John A. Burns School of Medicine, Honolulu, HI, United States; ^6^Department of Medical Biophysics, University of Toronto, Toronto, ON, Canada; ^7^Hurvitz Brain Sciences Program and Physical Sciences Platform, Sunnybrook Research Institute, University of Toronto, Toronto, ON, Canada; ^8^Department of Neurosurgery, Stanford School of Medicine, Stanford, CA, United States; ^9^Department of Bioengineering, Stanford University, Stanford, CA, United States

**Keywords:** mild TBI, concussion, MRI, sports, neuroanatomy, football

## Abstract

**Background and Purpose:** Athletes participating in high-contact sports experience repeated head trauma. Anatomical findings, such as a cavum septum pellucidum, prominent CSF spaces, and hippocampal volume reductions, have been observed in cases of mild traumatic brain injury. The extent to which these neuroanatomical findings are associated with high-contact sports is unknown. The purpose of this study was to determine whether there are subtle neuroanatomic differences between athletes participating in high-contact sports compared to low-contact athletic controls.

**Materials and Methods:** We performed longitudinal structural brain MRI scans in 63 football (high-contact) and 34 volleyball (low-contact control) male collegiate athletes with up to 4 years of follow-up, evaluating a total of 315 MRI scans. Board-certified neuroradiologists performed semi-quantitative visual analysis of neuroanatomic findings, including: cavum septum pellucidum type and size, extent of perivascular spaces, prominence of CSF spaces, white matter hyperintensities, arterial spin labeling perfusion asymmetries, fractional anisotropy holes, and hippocampal size.

**Results:** At baseline, cavum septum pellucidum length was greater in football compared to volleyball controls (*p* = 0.02). All other comparisons were statistically equivalent after multiple comparison correction. Within football at baseline, the following trends that did not survive multiple comparison correction were observed: more years of prior football exposure exhibited a trend toward more perivascular spaces (*p* = 0.03 uncorrected), and lower baseline Standardized Concussion Assessment Tool scores toward more perivascular spaces (*p* = 0.02 uncorrected) and a smaller right hippocampal size (*p* = 0.02 uncorrected).

**Conclusion:** Head impacts in high-contact sport (football) athletes may be associated with increased cavum septum pellucidum length compared to low-contact sport (volleyball) athletic controls. Other investigated neuroradiology metrics were generally equivalent between sports.

## Introduction

Repetitive head trauma commonly occurs in sports such as football, boxing, soccer, and mixed martial arts and can result in mild traumatic brain injury (mTBI), synonymous with concussion. The accumulation of multiple minor (i.e., sub-concussive) head injuries over many years could additionally contribute to long-term brain injury (1). Some studies have suggested that college football players average more than 14 significant head impacts per game (2, 3). Repetitive sports-related head impacts increase the long-term risk for developing neurodegenerative diseases, such as Alzheimer's disease, amyotrophic lateral sclerosis, and chronic traumatic encephalopathy (4–6). However, most mTBI patients have normal early qualitative imaging, with only 27% showing structural abnormalities, such as small hemorrhages (7). More sensitive neuroimaging techniques may detect the subtle anatomical and regional blood flow changes that may occur following mTBI (8). Recent quantitative neuroimaging has shown that high school and collegiate high-contact sports athletes, who experience multiple sub-concussive and concussive head impacts, show altered brain development (9–12), such as reduced cortical thinning over time (13).

Outside of high-contact sports, brain MRIs from patients with mTBI exhibit anatomic differences amenable to neuroradiologic interpretation, including cavum septum pellucidum (CSP) anatomy, prominence of perivascular and CSF spaces, white matter integrity, disrupted perfusion, and hippocampal volume. The CSP is an apparently normal-variant cavity bounded by the two leaves of the septum pellucidum, the corpus callosum, and the fornix (14). The CSP has a high prevalence in chronic traumatic encephalopathy (15), present in 65% of neuropathologically-confirmed cases (16). Subsequent studies have demonstrated the progressive development of a new CSP in boxers (17) and found that, in a cohort of 100 boxers, 43 had CSP, 32 had dilated perivascular (Virchow-Robin) spaces, and 19 had enlarged lateral ventricles (18). One study found a significantly higher number of dilated perivascular spaces in otherwise healthy patients with recent mTBI compared to controls (19). Enlargement of CSF spaces is a common finding in autopsy studies of chronic traumatic encephalopathy in athletes (16). Ventricular enlargement has likewise been observed in studies of repetitive mild head trauma in juvenile rats (20, 21). White matter hyperintensities are also a common finding in mTBI patients (22, 23). An arterial spin labeling (ASL) perfusion study reported significant reductions in cerebral blood flow in both bilateral and unilateral cortical structures in mTBI patients (24). Additionally, mTBI patients show multiple white matter regions with reduced fractional anisotropy (FA), potentially reflecting axonal injury (25). Finally, patients with a remote history of mTBI show reduced hippocampal volume later in life compared to age-matched controls (26).

In this study, we aimed to determine whether these neuroradiologic brain findings are more prevalent in football athletes compared to volleyball athletic controls. Additionally, we sought to determine whether, within the sport of football, these metrics varied with clinically relevant football experience (prior concussions, years of prior football, position-associated injury risk). This is intended to describe how clinical neuroradiology findings can be applicable to the study of sport.

## Methods

### Study Population

The study was introduced to college football and volleyball teams in conjunction with athletic trainers. Male volleyball athletes were chosen as athletic controls due to the low-contact play and low incidence of concussion (27). Exclusion criteria were self-reported history of brain surgery, severe brain injury, or major neurological, psychiatric, or substance abuse disorders. Sixty-three high-contact (football) and 34 low-contact (volleyball) athletes voluntarily provided written informed consent in accordance with the Stanford Institutional Review Board and Health Insurance Portability and Accountability Act. The median age for volleyball was 19.34 years (IQR 18.83–20.43), and the median age for football was 18.65 years (IQR 18.42–19.21) (*p* = 0.0007). All athletes underwent brain MRI at the start of their respective seasons, and after the last season of sports participation. In addition, 13 of the football athletes underwent post-concussion imaging (concussions were identified by athletic trainers). Each athlete had one to eight MRI time points spanning a maximum of 4 years, with a total of 315 MRI scans. Fifty-three football and (27) volleyball athletes had more than one scan, distributions shown in Supplementary Figure 10. A board- and Certificate of Added Qualification-certified neuroradiologist with 9 years of post-residency experience blindly examined all scans for incidental abnormalities, resulting in exclusion of three athletes (each with one, three, and five timepoints, respectively). Four volleyball athletes sustained a concussion during the study, and their post-concussion scans were also excluded (each with one, one, two, and three time points, respectively). After exclusions, 62 football and 32 volleyball athletes remained, with a total of 210 football and 89 volleyball longitudinal MRI scans (Table 1). No new neuroradiologic abnormalities were identified after concussions.

**Table 1:** Clinical metrics for the study population.

**Sport**	**# of athletes**	**Years of playing football**	**Presence of prior concussions**	**Baseline SCAT score**	**Position-based risk**
Football	62	Median 9, IQR 4, range 4–15	24	Median 55, IQR 5, range 42–61	Median 31, IQR 3.6, range 28.9–36.1
Volleyball	32	Median 0, IQR 0, range 0–2	2		

Additional information obtained for football players included the self-reported number and timing of past concussions, the number of years playing football, and the current position played. One football player did not provide years of contact football experience and was excluded from analyses involving years of prior football exposure. Football players completed a pre-first season Standardized Concussion Assessment Tool (SCAT) II evaluation, specifically the cognitive assessment, balance, coordination, and standardized assessment of concussion portions (maximum score 61, with lower scores corresponding to worse performance).

### Image Acquisition

Using a 3-Tesla scanner (GE MR 750; GE Healthcare, Milwaukee, WI) and an eight-channel receive head coil, we acquired:

- To assess brain volume, which is shown to be altered in mild TBI and concussion (13), as well as brain anatomy: T1-weighted axial; 3D inversion-recovery fast spoiled gradient-echo, 1 mm isotropic, repetition-time (TR) 7.9 ms, echo-time (TE) 3.1 ms, acquisition time 5.1 min.
- To assess hippocampal volumes, shown to be abnormal in TBI (28): High-resolution T2-weighted images perpendicular to the hippocampal long axis; coronal oblique fast spin-echo, 0.39 × 0.52 × 2 mm, 0.2 mm skip, TR 5,000–15,000, TE 95–109, echo train length 25, matrix 512 × 384, number of excitations (NEX) 2, 32–40 slices, acquisition time 4.4–5.8 min.
- To measure cerebral blood flow (CBF) to the brain, which is thought to be altered in concussion (29): Arterial spin labeling (ASL) perfusion images; axial 3D, field-of-view 24 cm, slice thickness 4 mm, NEX 3, 512 points, 8 arms, post-label delay 2 s.
- Diffusion tensor imaging (DTI), which can show microstructural alterations in TBI (30, 31): b = 800 s/mm^2^, 30 directions, 3 b = 0 s/mm^2^ images, 1.875 × 1.875 × 2 mm, TE 81 ms, TR 8000 ms.
- 3D susceptibility-weighted angiography multi-echo gradient-echo, which can show microhemorrhages that might be related to TBI (32): 1 mm isotropic, 5–8 echoes ranging from 5 to 40 ms.
- To depict white matter lesions, which are found in TBI but are non-specific (22, 23): T2-FLAIR; 2D, TR 9500 ms, TI 2300 ms, 0.47 × 0.47 × 5 mm, acquired for 142 (103 football, 39 volleyball) out of 299 MRIs in total (at baseline, 15 football and 12 volleyball).

We computed fractional anisotropy, trace and mean diffusivity images using FSL's dtifit. We computed CBF based on the scanner-provided difference and proton density images based on the formulation described by Alsop et al. (33).

### Image Interpretation

Three board-certified radiologists with additional Certificates of Added Qualification in neuroradiology (separate from the incidental finding neuroradiologist) with nine, seven, and seven years of post-residency experience reviewed each of the athletes' MRI scans. Raters were blind to subject identity and sport, but they viewed all of a subject's longitudinal scans simultaneously. Raters analyzed the anatomic images as well as reconstructed images from the DTI scan (isotropic diffusion-weighted images, fractional anisotropy, mean diffusivity, and b = 0 s/mm^2^ images) and scanner-generated CBF maps. Beforehand, raters performed and discussed the same blinded assessments on an independent dataset to establish agreement on the rating scales. After agreement was established on the rating scales, the raters viewed the guide sheet in the supplement to rate the scans from the study. Specifically, the raters compared their images with the images in the rating guide to find the best match.

**Figure 1:**
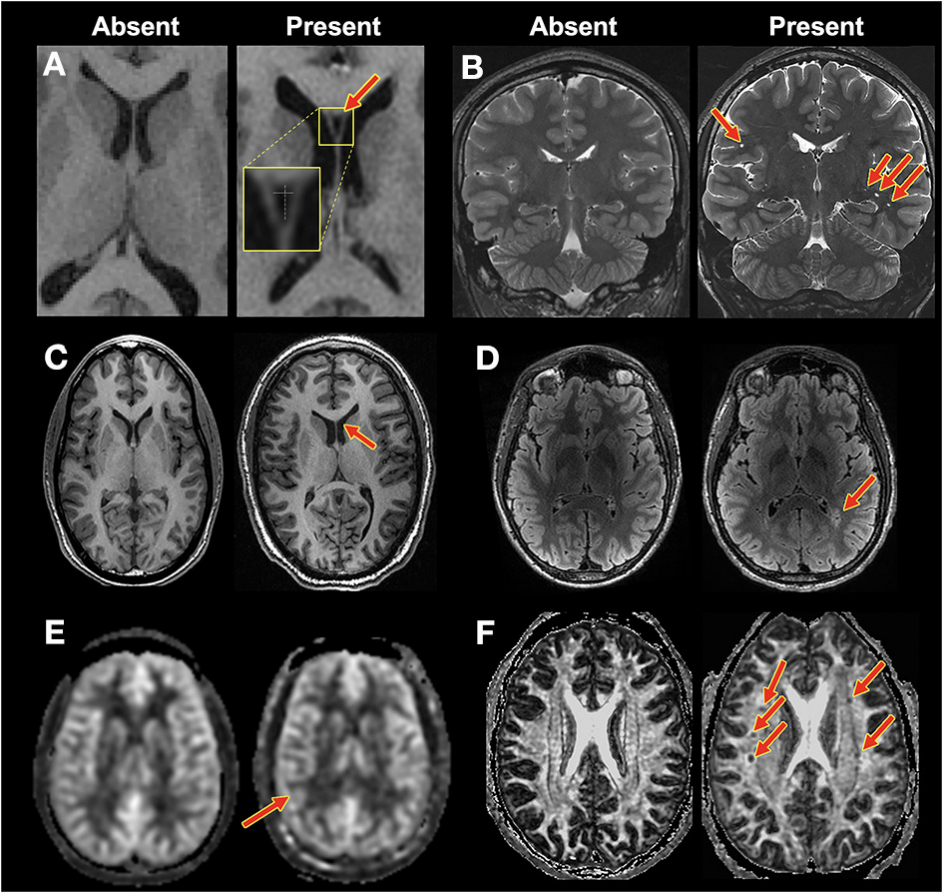
Example findings. Contrast for each image pair was as follows: **(A,C)**: T1-weighted. **(B)**: T2-weighted. **(D)**: T2-FLAIR. **(E)**: CBF. **(F)**: DTI: FA. Red arrows indicate findings. **(A)** CSP: normal (1) vs. CSP (2). **(B)** Perivascular spaces, none (1) vs. extensive (4), and hippocampal size, normal (1) vs. mild (2). **(C)** Extra-axial CSF spaces: normal (1) vs. moderate (3). **(D)** White matter hyperintensities: none (0) vs. severe (4). **(E)** CBF asymmetry: normal (1) vs. possibly asymmetric (2) **(F)** FA holes: none (1) vs. severe (4).

Raters assessed the items listed below (see Supplementary Material and Figure 1):

1. The morphological characterization of the CSP, termed “cavum extent.” (17, 18) (Supplementary Figure 1).
  1 = no detectable cavum.
  2 = small cavum anterior to the fornix.
  3 = cavum septum pellucidum et vergae.
  4 = cavum extending up to the fornix.
  5 = cavum vergae only.
  6 = cavum velum interpositum.
  No subjects met criteria 4–6.
2. The measured length and the width of the CSP if category ≥ 2 (Supplementary Figure 2).
3. The following features on a four-point scale, with 1 reflecting none or normal, 2 mild, 3 moderate, and 4 extensive or severe.
  - Number of perivascular spaces (PVSs) (18), defined as well-defined typically linear or ovoid T2 hyperintense foci ≥1 mm in diameter that suppress on trace/ADC images from DTI as well as T2 FLAIR (1 = none, 2 = mild, located along and near the inferolateral putamen; 3 = moderate, small T2 hyperintense foci, but not in or near the inferolateral putamen, typically in subcortical or periventricular white matter, <10 in number (18); 4 = extensive, same T2 hyperintense foci as in category 3, but >10 in number) (Supplementary Figures 3, 4).
  - Prominence of extra-axial CSF spaces (18) (1 = small ventricles and relatively narrow sulci, 2 = ventricle and sulci slightly larger and more prominent than expected for college age, with the frontal horns ~ < 1 cm in width, 3 = moderate, ventricles larger than expected for college age, and clearly in the prominent category, approximately a cm in width at the level of the frontal horns, 4 = marked, very large ventricles and sulci, greater than approximately one cm in width at the level of the frontal horns) (Supplementary Figures 5, 6).
  - The presence of white matter hyperintensities (WMH) (34, 35) in the cerebral white matter, defined as foci of T2 hyperintensity that do not fit the criteria for PVS above (1 = no foci of T2 hyperintensity in the supratentorial white matter, 2 = mild, few hyperintense T2 foci that are not CSF intensity perivascular spaces on FLAIR or trace/ADC images from the DTI, 3 or less in number; 3 = same, but from 4 to 10; 4 = extensive, >10).
  - CBF asymmetry (36) (2 = possibly asymmetric with subtly higher CBF on one side compared to the other, but < ~20%, 3 = probably asymmetric, with CBF ~20–30% higher, 4 = highly confident asymmetric, with CBF >30% higher on one side compared with the other) (Supplementary Figure 8).
  - Holes in the fractional anisotropy maps (30), which have primarily been studied quantitatively, though may be amenable to qualitative visualization (1 = no black dots in the white matter, 2 = black dots are present but overlap with perivascular spaces, 3 = black dots are present overlapping with perivascular spaces, and <3 do not overlap with perivascular spaces, 4 = same as category 3, except ≥3 that do not overlap with perivascular spaces). These non-overlapping foci could potentially relate to white matter injury, non-specific white matter spots, or perivascular spaces otherwise below detection limits (Supplementary Figure 9).
  - Left and right hippocampal size (37) (1 = normal, with hippocampal bodies and heads showing thick lamination and internal architecture, 2 = mildly small, with subtle prominence of the choroidal fissure and temporal horn along the superior aspect of the hippocampus, maximally ~2 mm, 3 = moderately smaller than expected for college age group, with a larger temporal horn and choroidal fissure on top of the hippocampus, maximally 5 mm, also including larger hippocampal sulcal remnants, 4 = markedly smaller than expected for age, with temporal horn/choroidal fissure measuring more than 5 mm) (Supplementary Figure 7).
4. The number of microhemorrhages detected (none were detected).

### Statistics

Rater agreement was assessed with Gwet's AC, a measure of categorical inter-rater variability (38). For the morphological characterization of the CSP, termed “cavum extent,” majority voting (2/3 raters) resulted in one rating per subject. Subsequent quantitative measurements (cavum length/width) were derived from the raters who agreed with the majority vote (for all subjects, at least two raters were in agreement). For all other metrics, we took the median across the neuroradiologists' ratings.

For each rating category with ordinal variables, we compared the baseline scans from the football and volleyball groups with a Wilcoxon-Mann-Whitney test. We corrected for multiple comparisons across these eight rating categories using a Bonferroni correction.

For quantitative metrics at baseline (cavum length and width), we used a Wilcoxon rank sum test, correcting for multiple comparisons with a Bonferroni correction.

To examine longitudinal changes, we utilized general linear models: to evaluate longitudinal sport-specific changes in cavum length, we utilized a general linear model with log transformation, clustering by subject identity. To examine longitudinal relationships for all other metrics, we used a general linear model without transformation, clustering by subject identity.

To evaluate if all metrics together could separate the sports at baseline, we performed ordered logistic regression on each metric, and then assessed the combined metrics by using seemingly unrelated estimation (39).

#### Within-Football Analyses

Next, we performed within-football analyses at baseline to test the association of these potential factors:

1. Years of prior football experience (using a Spearman correlation for cavum length/width, and a Wilcoxon rank sum test for all other ratings).
2. The presence/absence of prior concussion(s) before study enrollment (using a Wilcoxon rank sum test for cavum length/width, and a Fisher's exact test for all other ratings). Only two volleyball players had a prior concussion, so we did not perform a similar prior concussion analysis for volleyball.
3. Baseline SCAT score at enrollment (using a Spearman correlation for cavum length/width, and a Wilcoxon rank sum test for all other ratings).
4. Player position-based sub-concussive (2, 40) (HITsp) and concussion risk (41).

For these within-football analyses, the rating categories were binarized for the Wilcoxon rank sum test as follows: perivascular spaces >3, prominence of CSF spaces ≥2, white matter hyperintensities ≥2, hippocampal size loss ≥2, CBF asymmetry ≥2, FA holes ≥2.

A Bonferroni correction was employed across rating categories.

All statistics were computed using Stata15 (StataCorp).

## Results

### Rater Agreement

Although the approach and metrics used have a subjective component, the use of a guiding template (see Supplementary Material) resulted in a strong interrater agreement (Gwet's AC: 0.811–0.923) among the three raters for the cavum extent, number of perivascular spaces, prominence of CSF spaces, number of white matter hyperintensities, and hippocampal size (Table 2). Interrater agreement was lower for CBF asymmetry and FA holes (0.693 and 0.631, respectively).

**Table 2:** For baseline MRIs, interrater agreement and baseline comparison of football with volleyball across rater metrics by a Wilcoxon-Mann-Whitney test.

**Region of interest**	**Interrater agreement**	**Football # in each categoryVolleyball # in each category**	**Football-volleyball comparison**
Cavum extent	0.923	5/55/24/28/0	*p =* 0.30
# of perivascular spaces	0.829	0/19/33/100/14/16/2	*p =* 0.12
Prominence of CSF spaces	0.873	51/10/1/027/4/1/0	*p =* 0.83
White matter spots	0.811	45/15/1/127/5/0/0	*p =* 0.19
CBF asymmetry	0.693	19/26/11/610/13/8/1	*p =* 0.88
Fractional anisotropy holes	0.631	0/55/6/10/30/2/0	*p =* 0.42
Left hippocampal size loss	0.812	39/22/1/020/12/0/0	*p =* 0.98
Right hippocampal size loss	0.872	42/19/1/043/19/0/0	*p =* 0.88

*Number of participants in each category per sport are indicated. Categories: Cavum extent: (1) = no cavum, (2) = tiny cavum, (3) = cavum septum pellucidum. All other metrics: (1) = none/normal, (2) = mildly increased, (3) = moderate, (4) = extensive. None of the differences of these metrics survived a multiple comparison threshold of 0.00625*.

### Rating Comparison of Football vs. Volleyball Controls

At baseline, none of the metrics showed significant differences between football and volleyball controls (Table 2). The omnibus test distinguishing sports combining across metrics was not significant (*p* = 0.20). A general linear model comparing metrics over time between sports showed a slight decrease in CBF asymmetry in both cohorts (*p* = 0.031, which did not withstand multiple comparisons correction) without a distinction between cohorts over time (*p* = 0.147). All other metrics showed stability over time and between both cohorts (*p* > 0.05).

### Quantitative Comparison: Cavum Length and Width at Baseline

**Figure 2:**
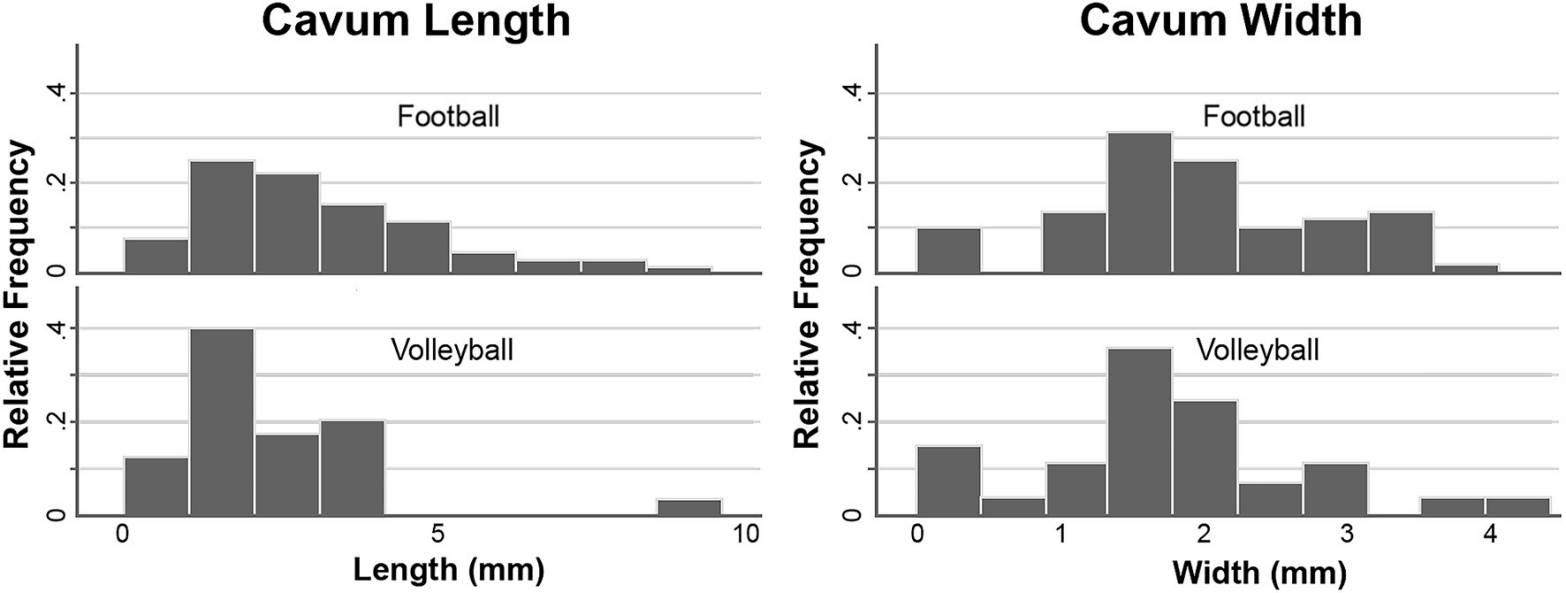
Histograms showing that the cavum length is greater in football compared to volleyball (left), while the cavum width is similar (right).

The baseline cavum length and width were assessed for athletes who had a category 1 or 2 cavum (category 3 had two subjects only in football). At baseline, cavum length was significantly greater for football than volleyball controls (Figure 2, football median 2.75 mm/IQR 2.38, volleyball median 2.01 mm/IQR 1.48, *p* = 0.02, survives Bonferroni correction). Inclusion of category 3 (*p* = 0.01) or exclusion of category 1 (*p* = 0.02) did not change the significance. A cavum length of <2 mm is 72% sensitive and 50% specific for discriminating football from volleyball. Cavum width was similar between college football compared to volleyball (football median 1.82 mm/IQR 1.15, volleyball median 1.70 mm/IQR 0.73, *p* = 0.38).

Since there was a difference in the cavum length at baseline, we evaluated this metric longitudinally using a general linear model, but there was no significant divergence between sports over time (*p* = 0.34).

We also studied the effect of head/brain size on the metrics: We found no difference in Freesurfer-estimated head size between sports (*p* = 0.85). Cavum length was not correlated with head size (*p* = 0.3). Non-parametric regression including head size as well as normalizing cavum length for head size did not change the significance of cavum length (*p* = 0.024 and 0.026, respectively).

### Within-Football Analyses at Baseline

#### Years of Playing Football

**Figure 3:**
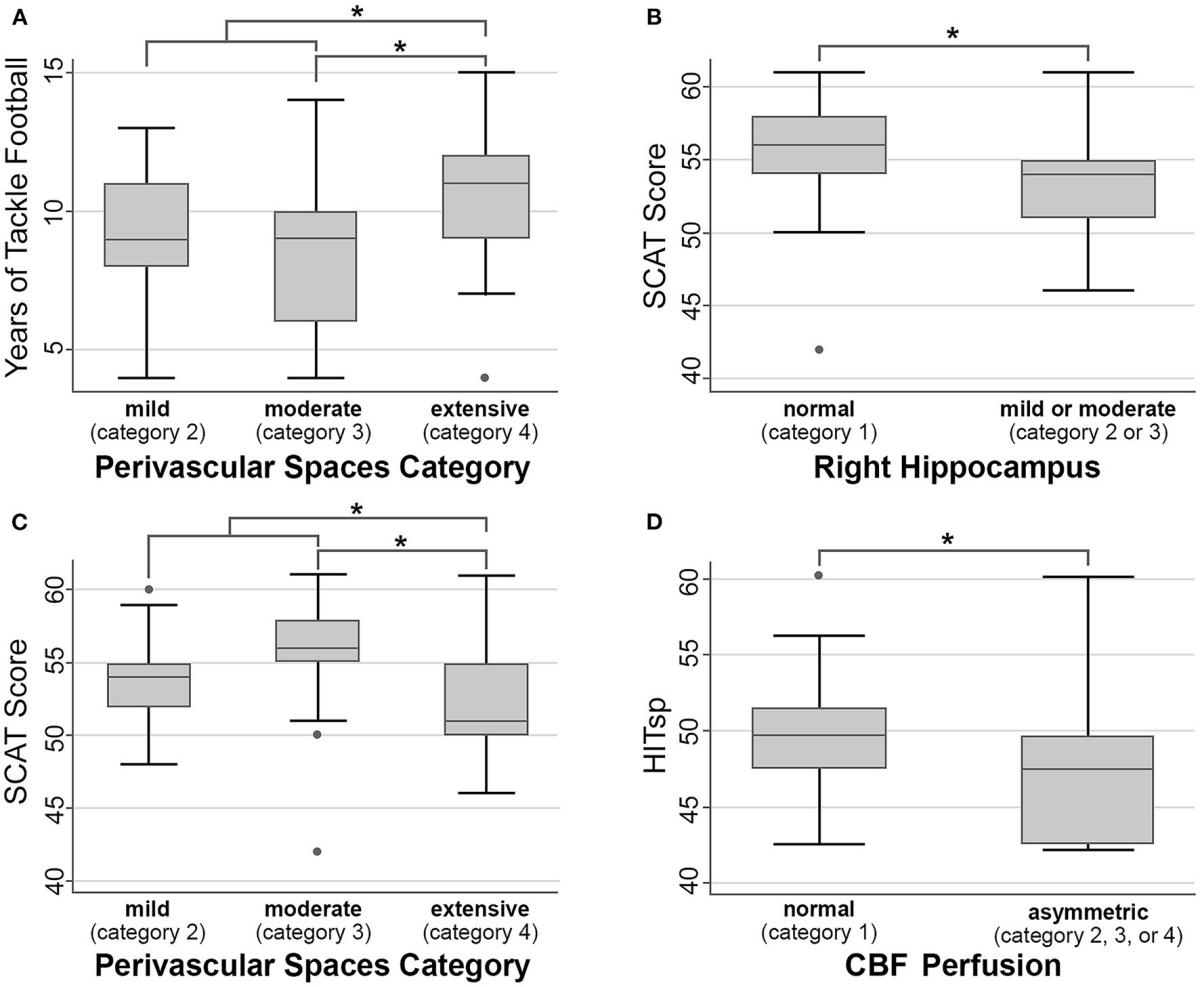
Within-football analyses. Asterisk (*) indicates *p* < 0.05 uncorrected, but none of these comparisons survive multiple comparison correction, and as such should be considered non-significant trends. **(A)** Years of tackle football vs. number of perivascular spaces. **(B,C)** SCAT score vs. right hippocampal size and number of perivascular spaces. **(D)** HITsp metric of sub-concussive risk and CBF perfusion.

Football athletes had between four and 15 years (median 9 years) of experience playing tackle football prior to their baseline MRI. The number of perivascular spaces evident on the baseline MRI was slightly higher with more years of prior football if one outlier was excluded from the extensive (4) category [comparing moderate (3) vs. extensive (4), *p* = 0.03 uncorrected, does not survive multiple comparison correction; comparing combined categories mild (2) and moderate (3) vs. extensive (4), *p* = 0.03, Figure 3A]. There were no significant differences associated with more years of prior football in cavum length (*p* = 0.79), width (*p* = 0.80), prominence of CSF spaces (*p* = 0.40), white matter hyperintensities (*p* = 0.77), CBF asymmetry (*p* = 0.22), FA holes (*p* = 0.34), left (*p* = 0.36) or right hippocampal size (*p* = 0.23).

#### Number of Prior Concussions

Within the football group, 24 of the 62 athletes had a history of concussion prior to baseline scanning (in comparison, only two out of 32 volleyball athletes had a history of concussion). There were no significant differences associated with the presence of prior concussions in football in cavum length (*p* = 0.35), width (*p* = 0.79), number of perivascular spaces (*p* = 0.29), prominence of CSF spaces (*p* = 0.83), white matter hyperintensities (*p* = 0.40), CBF asymmetry (*p* = 0.52), FA holes (*p* = 0.50), left (*p* = 0.33) or right hippocampal size (*p* = 0.87).

#### Baseline SCAT Scores

Athletes with more perivascular spaces had slightly lower baseline SCAT scores [comparing subjects rated moderate (3) vs. extensive (4), *p* = 0.02 uncorrected, does not survive multiple comparison correction; comparing combined categories mild (2) and moderate (3) vs. extensive (4), *p* = 0.04, Figure 3C]. Similarly, athletes with smaller right hippocampal sizes exhibited slightly lower SCAT scores [*p* = 0.02 uncorrected, does not survive multiple comparison correction, Figure 3B]. There were no significant differences associated with SCAT scores in cavum length (*p* = 0.28), width (*p* = 0.16), prominence of CSF spaces (*p* = 0.14), white matter hyperintensities (*p* = 0.27), CBF asymmetry (*p* = 0.88), FA holes (*p* = 0.70), or left hippocampal size (*p* = 0.08).

#### Position-Based Risk

There were no significant differences associated with position-based sub-concussion risk in cavum length (*p* = 0.99), width (*p* = 0.68), number of perivascular spaces (*p* = 0.78), prominence of CSF spaces (*p* = 0.23), white matter hyperintensities (*p* = 0.83), FA holes (*p* = 0.70), left (*p* = 0.69) or right hippocampal size (*p* = 0.09). However, CBF showed less asymmetry with a greater HITsp [Figure 3D, *p* = 0.01 uncorrected, does not survive multiple comparison correction]. There were no significant differences associated with position-based concussion risk in cavum length (*p* = 0.21), width (*p* = 0.33), number of perivascular spaces (*p* = 0.57), prominence of CSF spaces (*p* = 0.77), white matter hyperintensities (*p* = 0.43), CBF asymmetry (*p* = 0.80), FA holes (*p* = 0.33), left (*p* = 0.95) or right hippocampal size (*p* = 0.46).

## Discussion

In this study, we examined whether football athletes, compared to volleyball controls, exhibit neuroradiologic changes in multiple parameters: cavum septum pellucidum prevalence and dimensions, number of perivascular spaces, white matter hyperintensities, prominence of extra-axial spaces, hippocampal size, presence of microhemorrhages, CBF asymmetry, and holes in the FA maps. Overall, we found no significant difference between football athletes and volleyball controls for the neuroradiologic metrics (Table 2), suggesting that the presence of these findings is not specific to sports-related brain injury. While the cavum width was equivalent between sports, the cavum length was significantly greater among college football compared to volleyball athletic controls at baseline (median lengths of 2.8 and 2.0 mm, respectively). However, this finding had limited specificity for football. At baseline within football, we found several associations, but none of these met significance after multiple comparison correction. For example, a higher number of perivascular spaces showed a trend with more years of prior football experience. Also within this group, a lower SCAT score showed a trend with more perivascular spaces and smaller right hippocampal sizes. Athletes in positions with greater literature-referenced impacts to the head showed a trend of less CBF asymmetry. None of these trends survived multiple comparison correction, though they could suggest future avenues of quantitative investigation.

Studies assessing the occurrence of the CSP in high-contact sports date back to as early as 1963, with pneumoencephalography showing its presence in six out of nine boxers (42). In a large post-mortem study of 1,032 human brains (from non-TBI subjects), the age-dependent prevalence of CSP was 13% for men age 25–35 (43). Subsequent studies evaluating CSP in American football players observed CSP rates of 92% (44) and 94% (45) respectively, suggestive of a sports-related elevation. In comparison, a similar study evaluating boxers and mixed martial artists reported a rate of 53.1% compared to 17.7% in controls (46). While we did not find categorical differences in the CSP in our cohort, the greater CSP length may be potentially related to sport rather than congenital differences.

Our work can be considered in the context of the literature that associates high-contact sports with decreased brain volumes and neurocognitive measurements, and intends to present clinical neuroradiologic findings that can provide insight into high-contact sports' effects on the brain. A study by Bigler et al. showed the relationship between hippocampal atrophy and chronic diffuse TBI (47). Interestingly, more recent studies have reported smaller right hippocampal volumes after TBI in both children (48) and adults (49). Although we did not find significant differences in hippocampal size associated with more years of contact football exposure, the trend we observed of a median two point lower SCAT score accompanying a smaller visually-assessed right hippocampal size could possibly suggest a functional consequence of reduced right hippocampal volume. This parallels longitudinal quantitative hippocampal data showing progressive right hippocampal volume decreases in football compared to volleyball athletes from this same dataset (50). Furthermore, the presence of dilated perivascular spaces and their correlation with TBI has been shown by Inglese et al. in 2005 and 2006 and also by Orrison et al. in 2009, possibly indirectly reflective of brain volume loss (18, 19, 51). Possibly consistent with these findings, we observed a non-significant trend toward a greater number of perivascular spaces associated with more years of contact football exposure.

There are several limitations to this study. Neuroradiologic interpretation is inherently subjective, so our neuroradiologists reached rating agreement on an independent training set beforehand. Baseline MRI of the anatomic regions of interest prior to first exposure to high contact sports was not available, so we cannot exclude the possibility that some of these findings were present developmentally. With regard to white matter hyperintensities, (migraine history, which can be associated with FLAIR hyperintensities), was not available. Future longitudinal studies may follow athletes in youth tackle football with a baseline MRI prior to first exposure. This study was performed with male subjects from a single institution and has limited power for some of the smaller subgroup analyses (e.g., concussion), and future studies should include athletes of both sexes from multiple institutions. In this study, we correlated with literature-referenced frequency of position-based impacts, and future studies could similarly record and correlate MRI ratings with the total number and severity of measured impacts to the head using accelerometer-based technology (52). Volleyball was chosen as a low-impact control group, based on meta-analysis data showing that it has a very low concussion incidence rate compared to other sports (27). However, the fact that there were 4 concussions in this group during our study highlights the need for carefully selecting low-contact cohorts, preferably from multiple low-contact sports. Analysis of fractional anisotropy holes has generally been performed quantitatively (30), and our exploratory attempt at qualitative analysis did not prove fruitful. Detailed analysis of quantitative structural, diffusion, ASL and other metrics is beyond the scope of this study, but is the subject of our other published (13, 50) and future manuscripts.

## Data Availability Statement

Data supporting the conclusions of this article may be made available upon request.

## Ethics Statement

The studies involving human participants were reviewed and approved by Stanford University Institutional Review Board. Participants voluntarily provided written informed consent in accordance with the Institutional Review Board and Health Insurance Portability and Accountability Act.

## Author Contributions

GG, DC, MW, DD, DM, and MZ contributed to the conception and design of the study. BB, LM, DD, MG, and PD assisted in data acquisition. BB, LM, and ET performed neuroradiologic rating and analysis. MGo, MK, MGe, PD, NM, CA, and MZ contributed to data interpretation. JR assisted with the statistical analysis. DM, CA, and MZ wrote the manuscript and created figures. All authors contributed to manuscript revision, read, and approved the submitted version.

## Conflict of Interest

The authors declare that the research was conducted in the absence of any commercial or financial relationships that could be construed as a potential conflict of interest.

## Publisher's Note

All claims expressed in this article are solely those of the authors and do not necessarily represent those of their affiliated organizations, or those of the publisher, the editors and the reviewers. Any product that may be evaluated in this article, or claim that may be made by its manufacturer, is not guaranteed or endorsed by the publisher.

## Abbreviations

ASL: arterial spin labeling
CSP: cavum septum pellucidum
FA: fractional anisotropy
HITsp: position-based sub-concussion risk
mTBI: mild traumatic brain injury
SCAT: Standardized Concussion Assessment Tool.
