# Supplementary material for: Neuroradiologic Evaluation of MRI in High-Contact Sports
Source: Front Neurol. 2021 Aug 11;12:701948. doi: 10.3389/fneur.2021.701948 (PMC8385770; doi:10.3389/fneur.2021.701948)

Rating Scales (Supplementary Material)

Neuroradiologic Finding

<table>
<tr><td rowspan="2"></td><th colspan="3">Cavum Anatomy</th><th>Perivascular Spaces</th><th>Prominence of CSF Spaces</th><th>Number of White Matter Hyperintensities</th></tr>
<tr><td>Cavum extent</td><td>Cavum septum length (A-P) (mm)</td><td>Cavum septum width (left-right) (mm)</td><td></td><td></td><td>(If no FLAIR, then T2 hyperintense lesions in the WM, not black on DWI image)</td></tr>
<tr><th rowspan="4">Rating Categories</th><td>No cavum (0)</td><td colspan="2" rowspan="4"></td><td>None (1)</td><td>Normal (1) for "college age group"</td><td>Normal (1) for “college age group”</td></tr>
<tr><td>Small cavum anterior to fornix (1)</td><td>Mild (2)</td><td>Slightly more prominent (2)</td><td>Mild (2)</td></tr>
<tr><td>Cavum extending up to fornix (2)</td><td>Moderate (3)</td><td>Moderate (3)</td><td>Several (3)</td></tr>
<tr><td>Cavum septum pellucidum et vergae (3)</td><td>Extensive (4)</td><td>Marked (4)</td><td>Extensive (4)</td></tr>
</table>

Neuroradiologic Finding

<table>
<tr><td></td><th>CBF Asymmetry</th><th>FA Holes</th><th>Right, Left Hippocampal Size</th><th>Number of Microhemorrhages</th></tr>
<tr><th rowspan="4">Rating Categories</th><td>Highly confidently normal (1)</td><td>None (1)</td><td>Normal (1)</td><td rowspan="4">Microbleed should be QSM bright (in contradistinction to calcification, which should be QSM dark).

Please write series/image numbers.</td></tr>
<tr><td>Possibly abnormal (2)</td><td>Mild (2): in perivascular spaces</td><td>Mildly small (2) for college</td></tr>
<tr><td>Probably abnormal (3)</td><td>Moderate (3): in perivascular spaces and <3 within white matter spots</td><td>Moderately small for college (3)</td></tr>
<tr><td>Highly confidently abnormal (4)</td><td>Severe (4): diffuse >3 within white matter spots</td><td>Very small for college (4)</td></tr>
</table>

Supplementary Figure 1: **Cavum Anatomy**

If CSP is present, classify as:
Type 1: Anterior to the fornix
Type 2: Extending up to the fornix
Type 3: Extending into the cavum vergae, or just within vergae, or tiny anterior to fornix and large at the vergae
Type 4: Vergae only
Type 5: Cavum velum interpositum

Example of fenestrated cavum septum

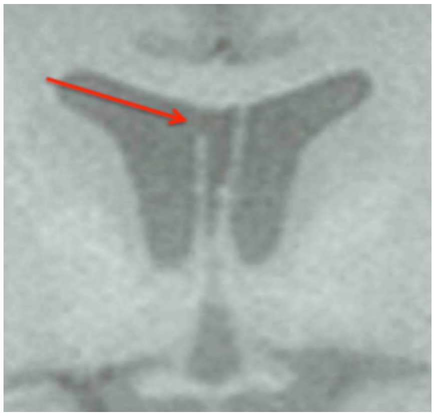

Normal (no CSP)

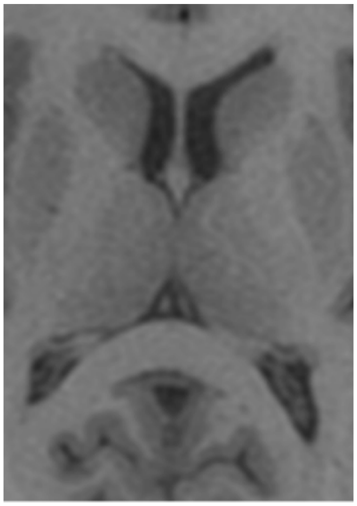

Type 1

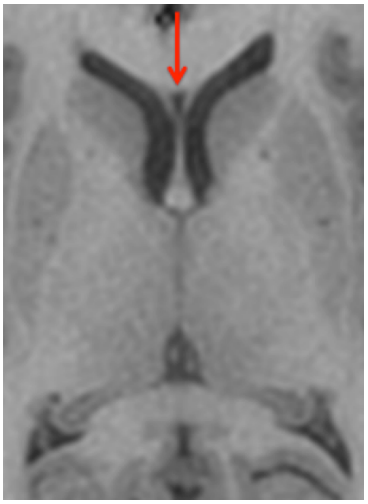

Type 2

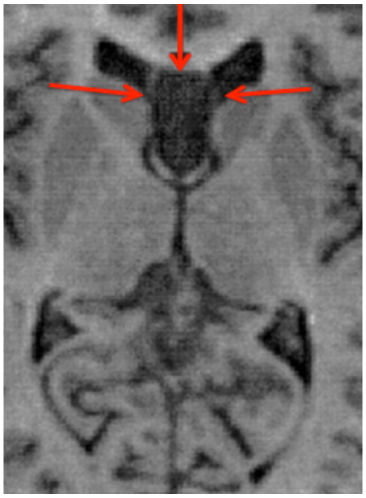

Type 3

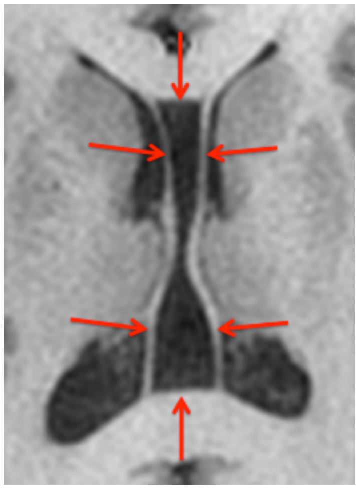

Type 4

Cavum vergae only.
Very rare without septum pellucidum (no examples).
Can distinguish from velum on sagittal images.

If observed: most likely velum interpositum.

Type 5

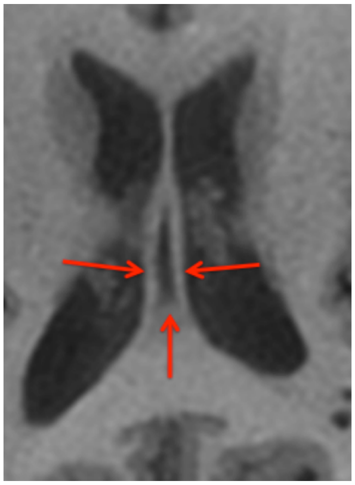

Cavum velum interpositum – must confirm on sagittal

Supplementary Figure 2: **Cavum Anatomy: Measurement**

Instructions: Measure maximum A-P and maximum transverse dimensions of the CSP in the axial plane of T1 BRAVO images. *If measuring vergae, pick a level at which the septum is still contiguous.*

Type 1

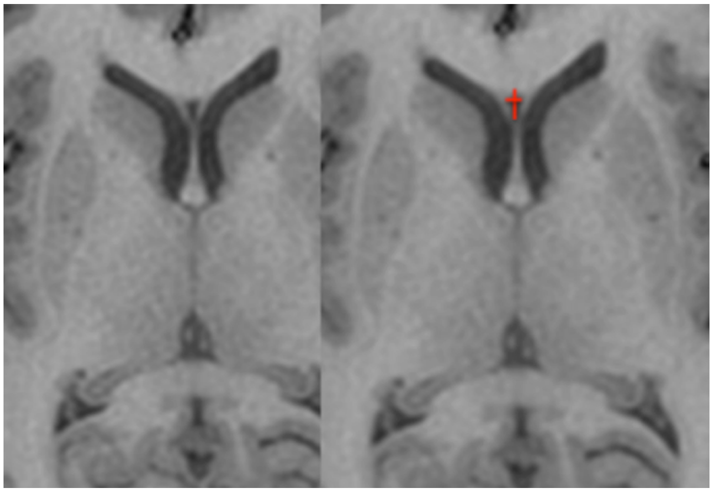

Type 2

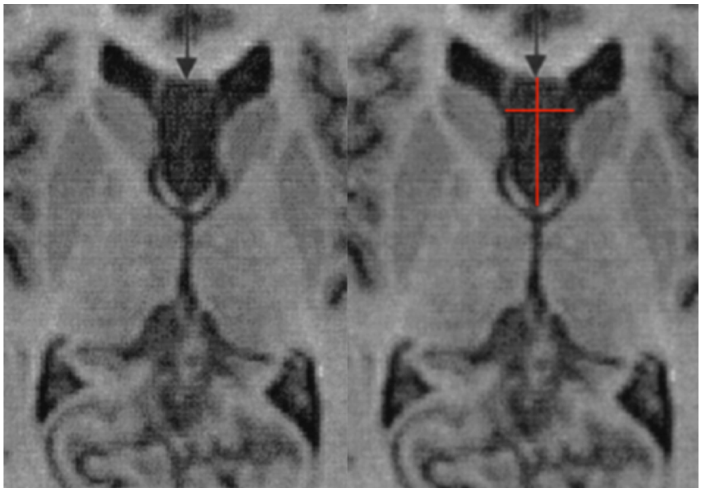

Type 3
(if wider anteriorly, measure anteriorly)

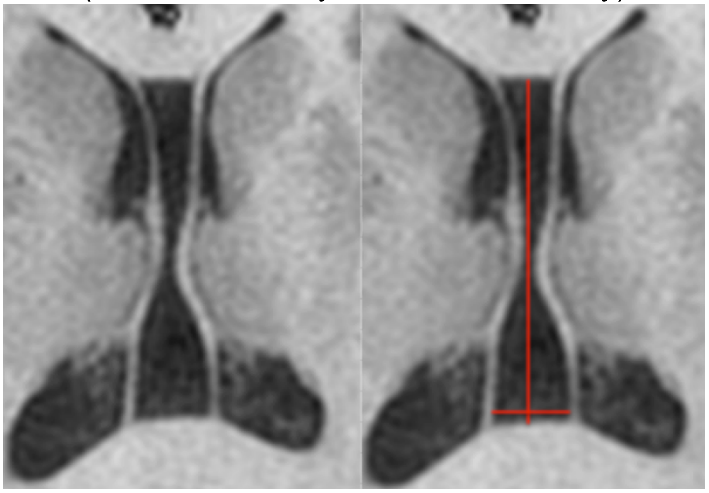

Cavum velum interpositum

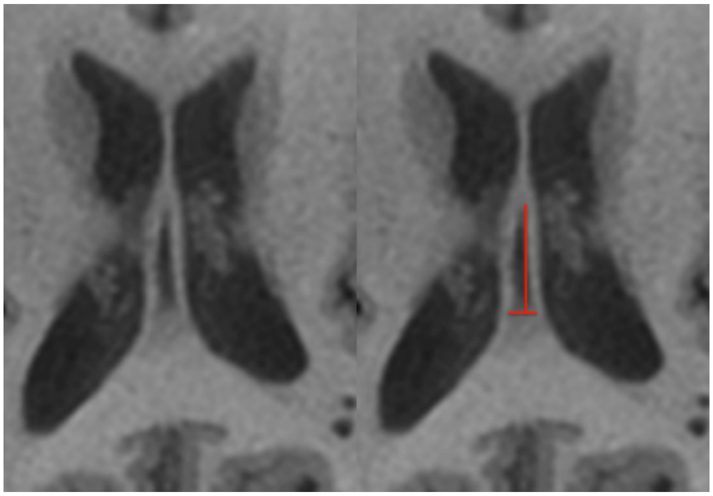

Supplementary Figure 3: **Perivascular Spaces**

Instructions: Count the number of perivascular (Virchow-Robin) spaces larger than 1 mm in the brain and assign a score (below). Use B0, coronal T2, DWI, ADC and T1 BRAVO images to determine the number of perivascular spaces.

Rating scale:
None (1)
Mild (2): In typical region of the inferior basal ganglia and thin linear in white matter, in any number
Moderate (3): Beyond the typical region of the inferior basal ganglia, thick/linear, < 10
Severe (4): Atypical location, thick and globular, excessive, > 10

None (1)

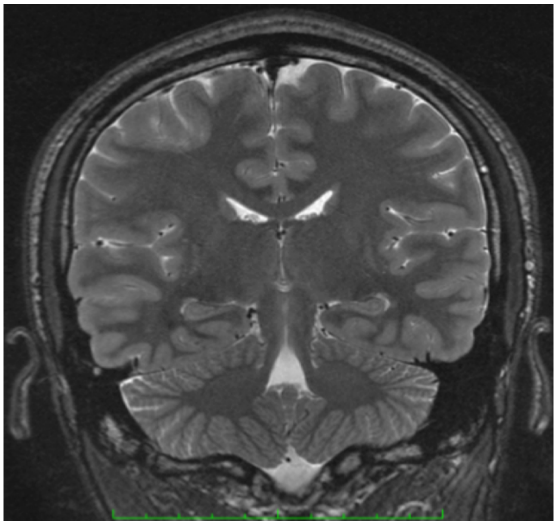

Mild (2)

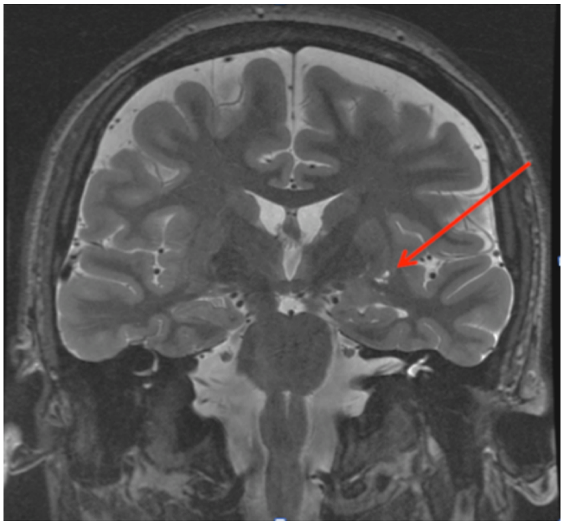

Moderate (3)

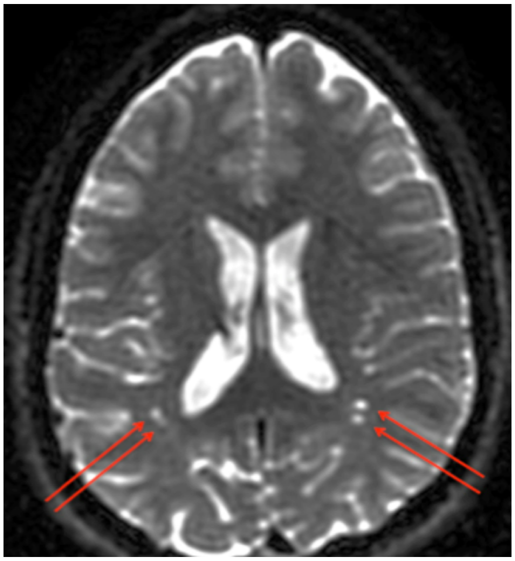

Severe (4)

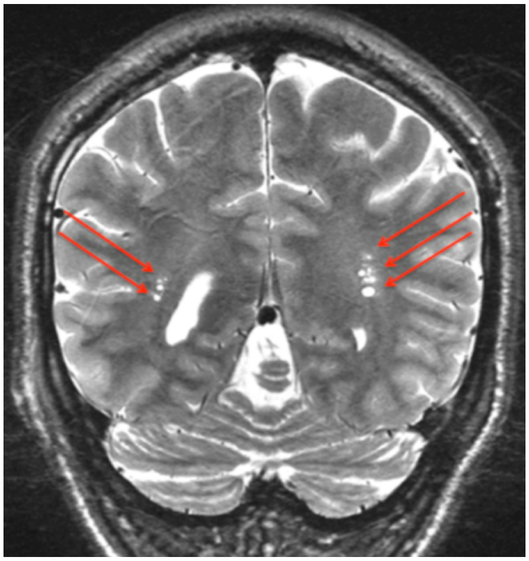

Supplementary Figure 4: **Perivascular Spaces (cont'd)**

Serial images of category 4 (severe) perivascular spaces:

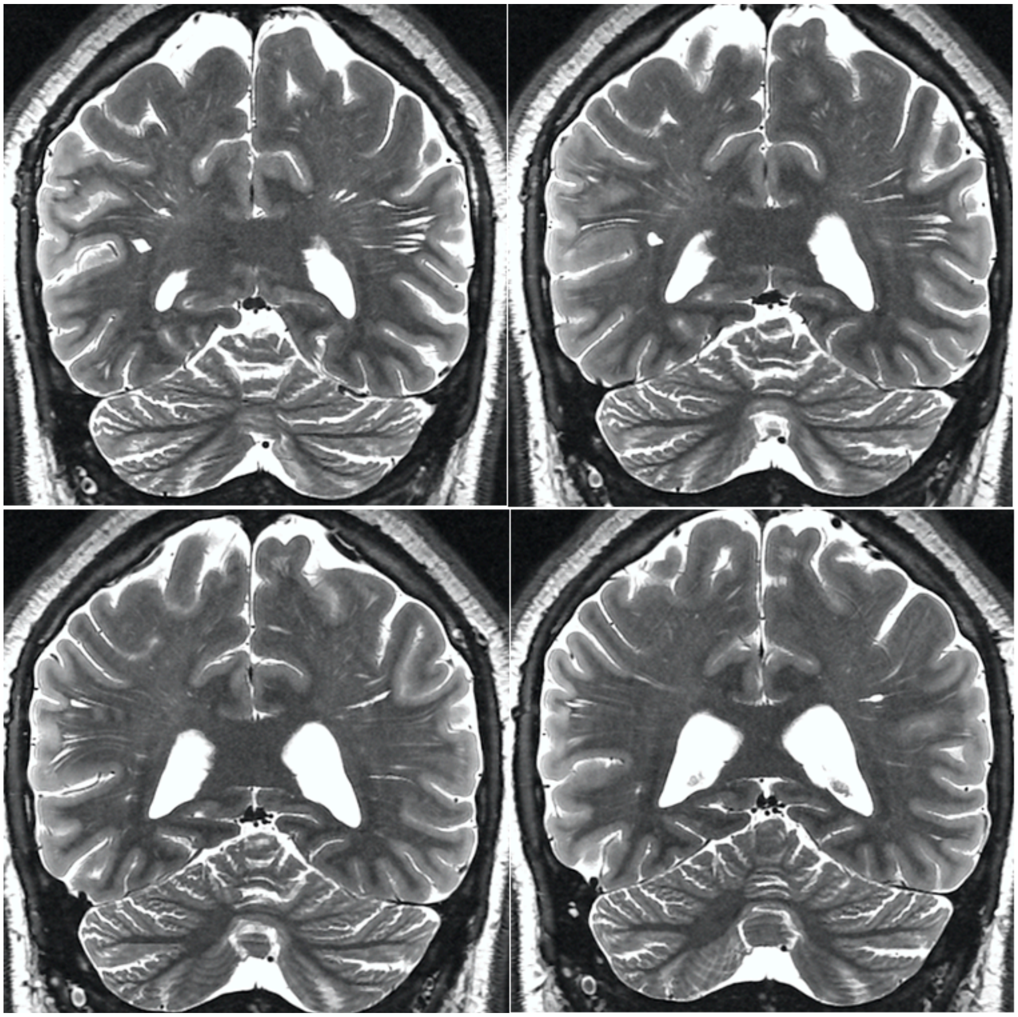

Supplementary Figure 5: **Prominence of CSF Spaces**

Instructions: Please consider both ventricular prominence and sulcal prominence when assessing CSF spaces.

Rating scale:
Normal (1): Normal for college age group
Mild (2): Slightly more prominent than expected for college age group
Moderate (3): Moderately more prominent than expected for college age group
Severe (4): Markedly more prominent than expected for age group

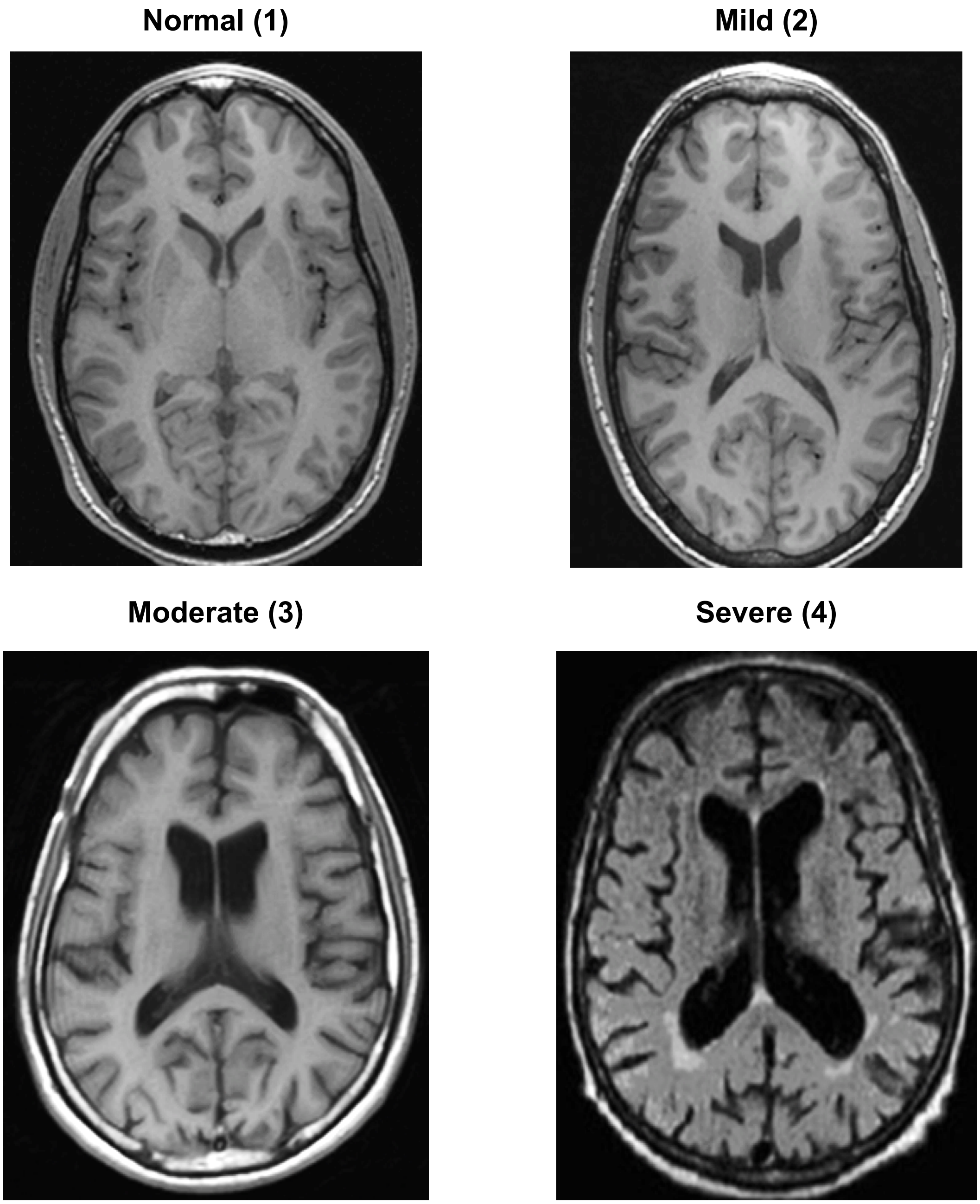

Supplementary Figure 6: **Prominence of CSF Spaces (cont'd)**

Sulcal and ventricular widening in the severe category:

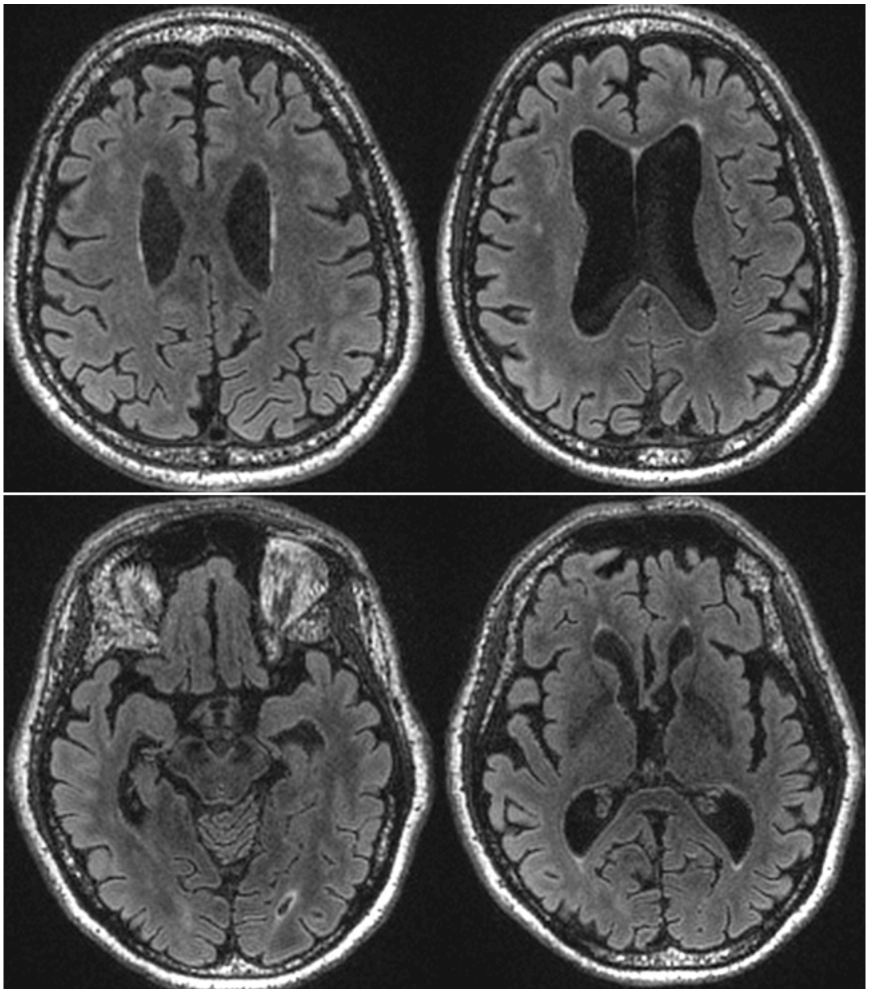

Supplementary Figure 7: **Hippocampal Size**

Rating scale:
Normal (1): Normal for college age group
Mild (2): Slightly smaller than expected for college age group
Moderate (3): Moderately smaller than expected for college age group
Severe (4): Markedly smaller than expected for age group

Normal (1)

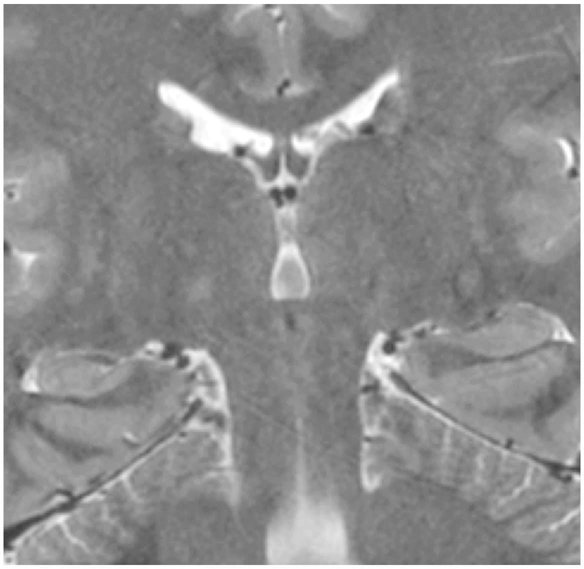

Mild (2)

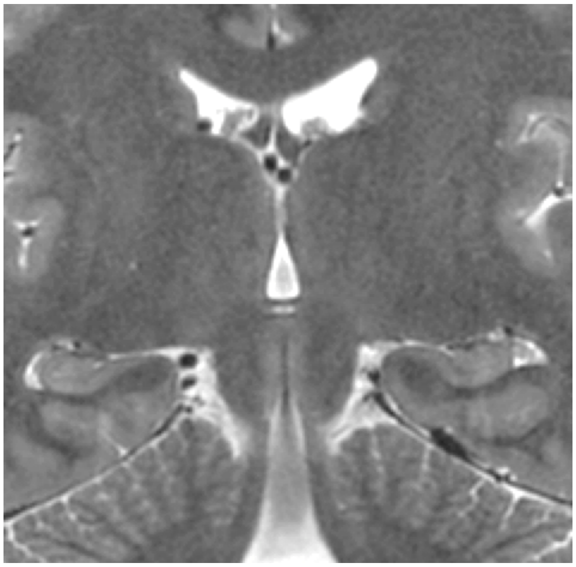

Moderate (3)

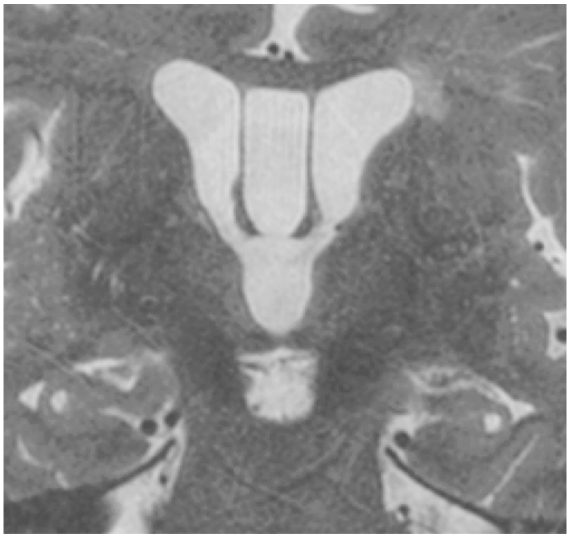

Severe (4)

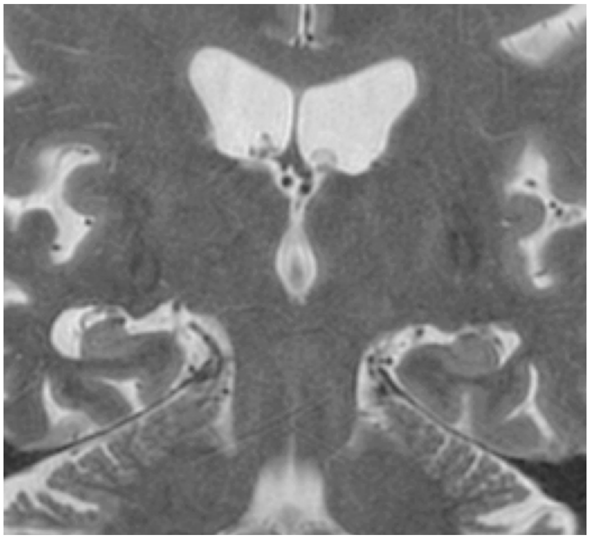

Supplementary Figure 8: **CBF Asymmetry**

Rating scale:
Highly confidently normal, symmetric (1)
Possibly abnormal (2)
Probably abnormal (3)
Highly confidently abnormal, asymmetric (4)

Normal (1)

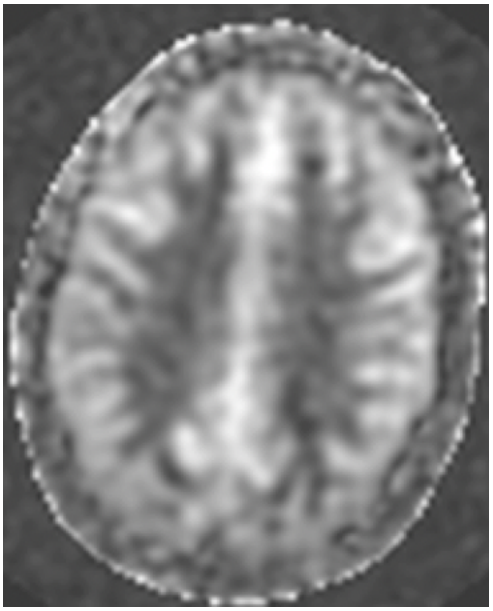

Possible asymmetry (2)

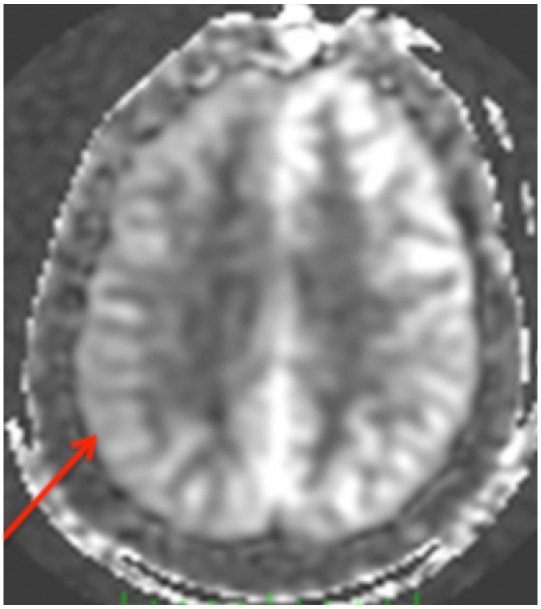

Probable asymmetry (3)

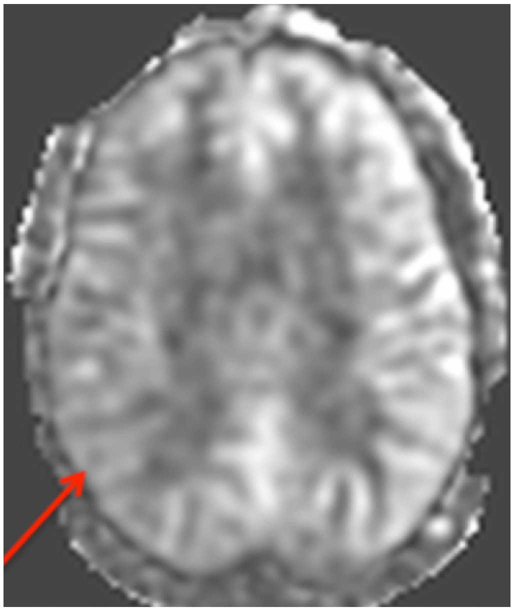

Definite asymmetry (4)

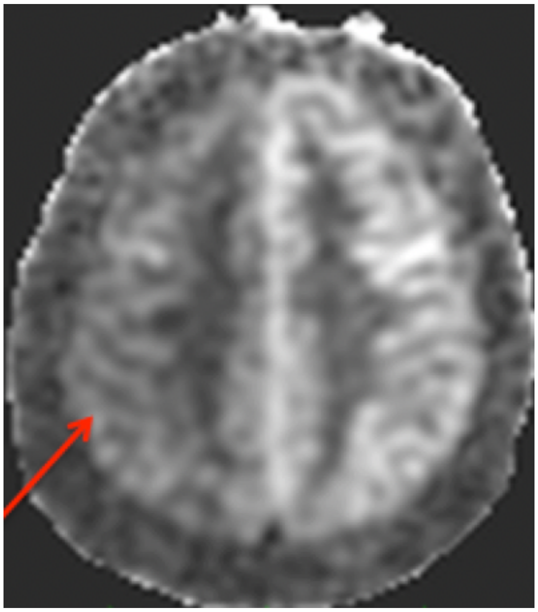

Supplementary Figure 9: **FA Holes**

FA holes seen on left image. Some of the larger ones (most posterior radiologic convention right) are apparent on the coplanar FLAIR as a perivascular space.

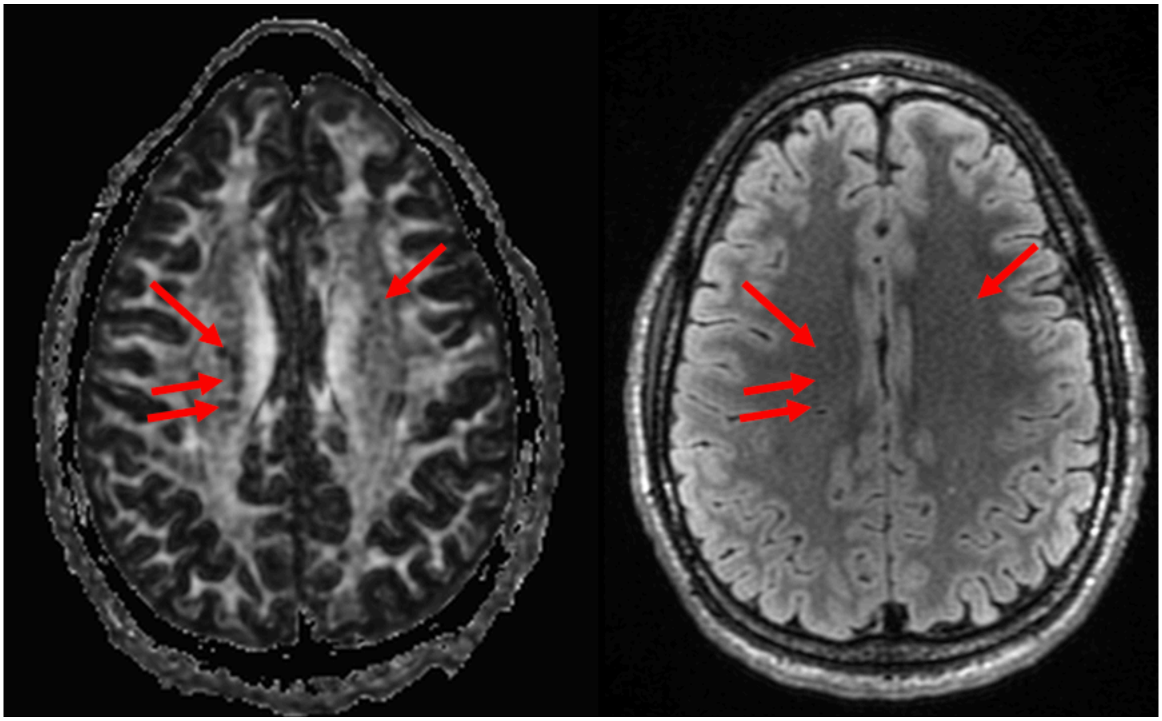

Supplementary Figure 10: **Demographic Information**

Distributions of football and volleyball athletes with more than one scan.

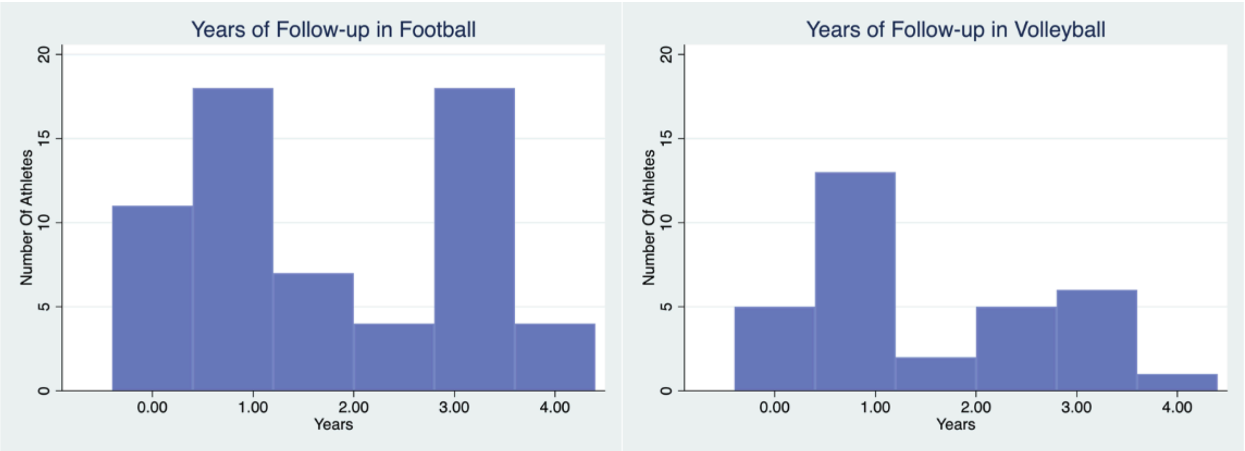

Supplement: Supplementary file 1 [file Data_Sheet_1.PDF]
